# Free functional muscle transfer after oncologic extremity reconstruction: A systematic review

**DOI:** 10.1016/j.jpra.2026.04.022

**Published:** 2026-04-30

**Authors:** Liahm Blank, Joshua Khorsandi, Hailey Kim, Sierra Adkins, Kyla Buttenberg, Demitri Franzoni, Joshua MacDavid

**Affiliations:** a Kirk Kerkorian School of Medicine at the University of Nevada, Las Vegas, 625 Shadow Ln, Las Vegas, NV 89106, USA; b Department of Plastic and Reconstructive Surgery, University of Nevada Las Vegas, Las Vegas, NV 89106, USA

**Keywords:** Free functional muscle transfer, Limb salvage, Extremity sarcoma, Functional reconstruction

## Abstract

**Background:**

Limb-salvage surgery for extremity sarcoma increasingly prioritizes preservation of meaningful motor function, yet oncologic resections often require removal of major musculotendinous units, resulting in severe disability despite limb preservation. Free functional muscle transfer (FFMT) offers a reconstructive option capable of restoring active motion through microvascular transfer and reinnervation of a donor muscle.

**Methods:**

A PRISMA-guided, OSF-registered systematic review was performed to evaluate outcomes of FFMT following oncologic extremity resection. Primary endpoints were flap survival and functional strength recovery (MRC/MMT), with secondary outcomes including complications and tumor recurrence. Outcomes were synthesized descriptively using pooled proportions with Wilson 95% confidence intervals.

**Results:**

Twenty-two studies comprising 82 cases met inclusion criteria. Flap survival was reported in 77 cases, with 100% survival (77/77; 95% CI 95.3–100.0). Complication outcomes were available in 60 cases, with a pooled complication rate of 26.67% (16/60; 95% CI 17.1–39.0%), most commonly involving wound-related events. Strength outcomes were most frequently in the MRC/MMT >3 to ≤4 range (36/82; 43.9%), while 15.9% achieved >4 to ≤5 strength. Mean strength varied by donor muscle, with vastus lateralis (4.21) and rectus femoris (4.17) demonstrating higher averages than latissimus dorsi (3.55) and gracilis (3.60), though subgroup sample sizes were small. Tumor recurrence was uncommon among reported cases.

**Conclusions:**

FFMT demonstrates high flap reliability and frequently meaningful strength recovery following oncologic extremity resection, supporting its role as a cornerstone technique for functional limb salvage. However, the evidence base remains limited by small heterogeneous series and incomplete outcome reporting, underscoring the need for standardized functional endpoints and prospective multicenter data collection.

## Introduction

Over the past several decades, the management of extremity soft-tissue sarcoma has shifted from routine amputation toward limb-salvage surgery and radiotherapy, reflecting the modern imperative to preserve not only limb length but meaningful postoperative function.^1^ Yet, “limb preservation” can be misleading when oncologic margin requirements mandate compartmental or en bloc excision of major musculotendinous units and/or adjacent neurovascular structures: even with an intact limb, patients may sustain profound disability if key motor functions such as elbow flexion, knee extension, or ankle dorsiflexion are lost.^2^ In contemporary reconstructive oncology, bringing in well-vascularized tissue is emphasized to improve reliability of healing, and preoperative planning is increasingly expected to anticipate not only coverage needs but also the feasibility of restoring functional loss.^3^

Free functional muscle transfer (FFMT) addresses this unique reconstructive problem by serving to replace missing motor units rather than only provide coverage of resected areas. Conceptually, FFMT involves the transfer of a functioning donor muscle with its neurovascular pedicle to a new anatomic location so that it can be revascularized and reinnervated to assume an independent mechanical role.^1^, ^2^, ^3^ In oncologic limb salvage, this indication is most compelling when resection eliminates active motion across a critical joint—such as loss of the anterior leg compartment producing foot drop—where FFMT can simultaneously resurface dead space and reanimate function.^3^^,^^4^

Donor selection and inset strategy are dictated by the target motion, defect geometry, and available recipient nerves/vessels. Across oncologic series and reviews, commonly utilized donor muscles include the latissimus dorsi and gracilis, with additional reported use of tensor fasciae latae and vastus lateralis depending on functional requirements and flap geometry. Recipient motor targets are similarly heterogeneous, ranging from femoral or sciatic-derived motor branches in the thigh to median-nerve motor fascicles in the forearm.^2^, ^3^, ^4^ Specific muscle neurovascular anastomosis selection is further compounded by patient selection for FFMT, as age, comorbidities (e.g., obesity, smoking, and cardiovascular disease), physical rehabilitation adherence, and the presence of suitable anatomy all contribute to success of the procedure.^4^^,^^5^

Despite increasing recognition that functional reconstruction is central to quality limb salvage, the oncologic FFMT evidence base remains dominated by case reports and small series, reflecting both the rarity of the indication and the variability in tumor location, extent of resection, and reconstructive strategy.^1^ The limited number of existing reviews combine analyses for pedicled and free functional muscle transfers^2^ or focus on free flap transfers following different pathologies, such as brachial plexus lesions.^6^ However, free functional muscle transfers following oncological resection are complex procedures that lead to unique challenges for surgeons (e.g., reconstructing nerves in tissues affected by radiation/chemotherapy), and thus should be examined independently.^1^

In this context, we present a PRISMA-guided systematic review focusing on patients undergoing major oncologic extremity resections requiring functional limb salvage and reconstruction with free functional muscle transfer (e.g., functional gracilis and latissimus dorsi). By systematically summarizing flap survival, functional strength outcomes, and complications, and by explicitly accounting for reporting completeness, we aim to provide pragmatic, clinically interpretable evidence for reconstructive decision-making in this high-impact setting.

## Methods

### Protocol and reporting

We conducted a prespecified, PRISMA-2020-conformant systematic review. The protocol was prospectively registered on the Open Science Framework (OSF; https://osf.io/5hb96). Eligible studies described patients undergoing major oncologic extremity resections requiring limb salvage reconstruction with free functional muscle transfer (FFMT). Our primary outcomes were flap survival and strength recovery; secondary endpoints included complications and tumor recurrence. Given expected heterogeneity and small sample sizes, outcomes were summarized descriptively rather than through formal comparative meta-analysis.

### Information sources and search strategy

**Figure 1:**
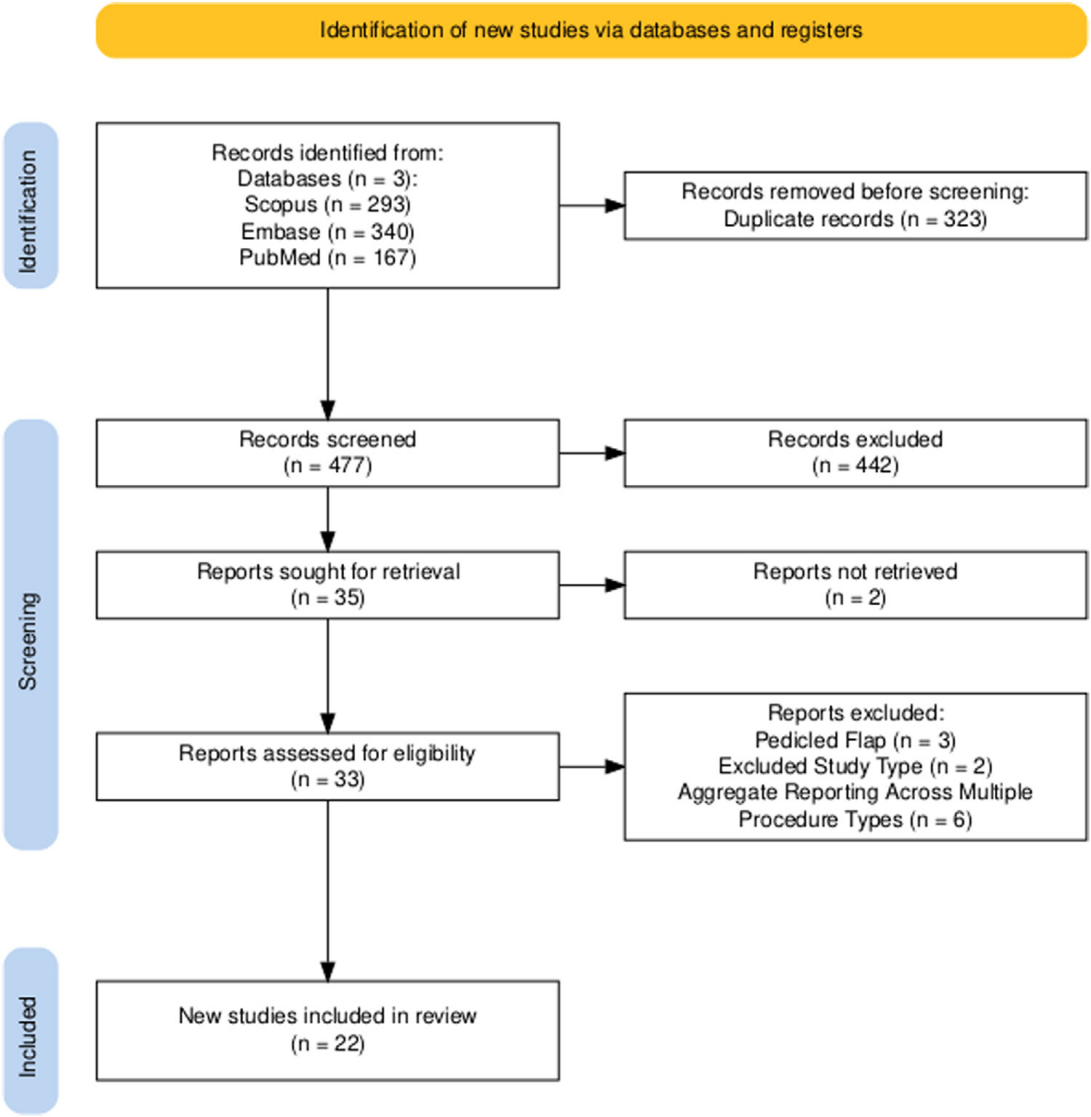
PRISMA flow diagram summarizing study identification, screening, exclusion, and inclusion for the systematic review (*n* = 22 included studies).

We systematically searched PubMed, Embase, and Scopus from database inception through December 15th, 2025. A representative PubMed string was: (“free muscle transfer” OR “functional muscle transfer” OR “free functional muscle” OR “functional free muscle” OR “free functional gracilis” OR “free gracilis” OR “free latissimus dorsi” OR “latissimus transfer” OR “functional gracilis” OR “functional latissimus” OR FFMT OR “reinnervated free flap” OR “neurotized free flap”) AND (sarcoma OR “soft tissue sarcoma” OR malignan* OR cancer* OR neoplasm* OR tumor* OR oncologic* OR “oncologic resection”)) NOT (cadaver* OR animal*). Equivalent search strategies were adapted for Embase and Scopus using database-specific syntax. Cross-database searching identified 800 records (Scopus *n* = 293, Embase *n* = 340, PubMed *n* = 167), of which 323 duplicates were removed, leaving 477 records for screening. Following title/abstract screening, 35 reports were sought for retrieval and 33 full-text articles were assessed for eligibility. Ultimately, 22 studies (82 cases) met inclusion criteria, with full-text exclusions due to non-oncologic FFMT cases (*n* = 3), excluded study type (*n* = 2), or aggregate reporting across multiple procedure types (*n* = 6) (see Figure 1).

### Eligibility criteria

Studies were included if they reported clinical human cases of upper- or lower-extremity oncologic resections requiring functional limb-salvage reconstruction and described reconstruction using a free functional muscle transfer. Studies were required to report at least one extractable outcome related to flap survival, complications, tumor recurrence, or strength recovery.

We excluded studies limited to pedicled muscle transfers, free flaps performed solely for soft-tissue coverage without functional intent, nerve transfers without muscle transfer, tendon transfers without muscle transfer, and animal or cadaveric studies. We also excluded studies regarding FFMT performed for trauma, congenital conditions, or chronic nerve injury unless clearly performed in the setting of tumor resection. Studies reporting aggregate outcomes across heterogeneous reconstructive procedures were excluded if FFMT-specific results could not be separated.

### Study selection and data collection

All records were imported into Rayyan for deduplication and staged screening (title/abstract followed by full-text review). Title and abstract screening were performed independently by two medical student researchers (H.K. and S.A.), trained in predefined inclusion and exclusion criteria, with substantial inter-reviewer agreement (Cohen’s κ = 0.67). Discrepancies were resolved through discussion, with a third medical student researcher (L.B.) adjudicating when needed. Full-text screening followed the same process, with moderate agreement (κ = 0.58), and reasons for exclusion were recorded.

Data extraction was performed collaboratively by H.K., S.A., and L.B. using a standardized spreadsheet at both study and case levels. Extracted variables included study characteristics, patient and tumor features, surgical and reconstructive details (donor muscle, nerve coaptation, recipient vessels), and outcomes (flap survival, complications, recurrence, and strength recovery). Physician investigators (J.M. and D.F.) provided methodological and clinical oversight throughout.

### Outcomes and definitions

Flap survival was defined as absence of total flap loss. Functional outcomes were extracted as Medical Research Council Scale for Muscle Strength (MRC) or Manual Muscle Testing (MMT) strength grades; an MRC/MMT score of 0 represents a lack of muscle contraction, while a score of 5 represents normal muscle contraction power.^7^ Strength recovery data from the few studies only reporting outcomes in Disabilities of the Arm, Shoulder, and Hand (DASH) or Musculoskeletal Tumour Society Score (MSTS) values were not included in pooled strength grade analyses.

Any complication was recorded as a binary endpoint (yes/no) when explicitly reported, and specific complications (e.g., infection, fracture, lymphedema, delayed wound healing) were captured when extractable. Tumor recurrence after resection was extracted when available as a binary endpoint (yes/no).

### Synthesis methods

Outcomes were synthesized descriptively due to heterogeneous case studies/series and incomplete reporting. Endpoints were summarized as proportions with Wilson 95% confidence intervals using reported numerators/denominators; analyses were limited to explicitly reported outcomes, with missing data coded as “not reported.” Strength outcomes (MRC/MMT) were summarized using descriptive statistics (mean, range, 95% CI) when available, with qualitative outcomes reported narratively. No formal meta-analysis, heterogeneity testing, or meta-regression was performed, and reporting completeness was calculated at both study and case levels.

### Risk of bias and certainty

Most included studies were small single-center case reports or retrospective series (Level IV). Therefore, formal risk-of-bias tools were not applied, and study quality was assessed primarily by reporting quality and completeness. Certainty of evidence was considered very low due to heterogeneity and selective reporting, and findings were interpreted descriptively.

## Results

### Study selection and yield

A total of 800 records were identified across databases (Scopus *n* = 293, Embase *n* = 340, PubMed *n* = 167). After removal of 323 duplicates, 477 unique records underwent screening, and 22 studies (82 cases) met inclusion criteria (Table 1). Reasons for full-text exclusion (*n* = 11) included pedicled flap procedures (*n* = 3), excluded study type (*n* = 2), and aggregate reporting across multiple procedure types without stratified FFMT data (*n* = 6).

**Table 1:** Summary of free functional muscle transfers after oncological resection. One row per article.

Citation	Included cases	Sex (M/F)	Mean age	Prior radiation therapy or chemotherapy (Yes/No)	Location of sarcoma (Upper extremity/Lower extremity)	Donor muscle used	Recipient nerve used
Carolina et al. (2025)^8^	1	0/1	59	0/1	L = 1	LD (1)	TN (1)
Brunetti et al. (2025)^9^	8	4/4	68	8/0	L = 8	VL (8)	FN (8)
Hoteit et al. (2025)^10^	1	1/0	61	1/0	L = 1	LD (1)	FN (1)
Brunetti et al. (2024)^11^	8	NR (8)	NR (8)	8/0	L = 8	VL (5), LD (2), RF (1)	FN (7), SN (1)
Bitoiu et al. (2022)^12^	1	1/0	35	1/0	U = 1	GL (1)	MN (1)
Lo (2022)^13^	1	1/0	56	1/0	L = 1	LD (1)	FN (1)
Nguyen et al. (2019)^14^	1	0/1	22	1/0	L = 1	GL (1)	FN (1)
Walley et al. (2017)^15^	3	1/2	54	2/1	L = 3	VL (3)	FN (3)
Nicoli et al. (2015)^16^	1	1/0	33	NR (1)	U = 1	LD (1)	RN (1)
Muramatsu et al. (2011)^17^	14	7/7	53	11/3	L = 14	LD (14)	FN (14)
Moschella et al. (2010)^18^	1	1/0	72	NR (1)	L = 1	RF(1)	TN (1)
MacHens et al. (2005)^19^	1	1/0	48	0/1	L = 1	LD (1)	NR (1)
Willcox et al. (2003)^20^	1	1/0	21	1/0	L = 1	LD (1)	FN (1)
Frey and Giovanoli (2003)^21^	2	2/0	30	NR (2)	U = 1, L = 1	LD (2)	RN (1), FN (1)
Hattori et al. (2001)^22^	1	1/0	20	NR (1)	U = 1	GL (1)	RN (1)
Doi et al. (1999)^23^	17	8/9	43	9/8	U = 2, L = 15	LD (12) GL (3) TFL (1) RF(1)	FN (7), SN (6), DPN (2), AN (1), RN (1)
Doi et al. (1997)^24^	2	0/2	26	0/2	U = 1, L = 1	GL (2)	RN (1), DPN (1)
Houdek et al. (2021)^25^	12	9/3	56	12/0	L = 12	LD (12)	SN (7), FN (5)
Lo et al. (2022)^26^	3	3/0	51	2/1	L = 3	LD (1), RF (1), VL (1)	FN (2), NR (1)
Huayllani et al. (2020)^27^	1	1/0	71	1/0	L = 1	LD (1)	FN (1)
Ihara et al. (1997)^28^	1	1/0	60	NR (1)	U = 1	TFL (1)	AN (1)
Ihara et al. (2000)^29^	1	1/0	62	NR (1)	L = 1	LD (1)	SGN (1)

Abbreviations: LD, Latissimus dorsi muscle; VL, Vastus lateralis muscle; GL, Gracilis muscle; RF, Rectus femoris muscle; TFL, Tensor fasciae latae muscle; NR, Not Reported; U, Upper extremity; L, Lower extremity; TN, Tibial nerve; FN, Femoral nerve; SN, Sciatic nerve; RN, Radial nerve; MN, Median nerve; DPN, Deep peroneal nerve; AN, Axillary nerve; SGN, Superior gluteal nerve; NR, Not Reported.

### Characteristics of the evidence base

The included studies were published between 1997 (inception) and 2025, with a median publication year of approximately 2016. Most reports consisted of single case descriptions or small case series, with a median of one case per study (IQR 2.75), a mean of 2.83 cases, and a maximum of 17 cases in any individual report. At the case level, the median patient age was 55 years (IQR 25), with a mean age of 50 years (range 9–82). Sex was reported in 75 of 82 cases; 56.1% were male (46/82), 35.4% were female (29/82), and sex was not reported in 8.5% (7/82). Geographically, studies originated predominantly from Japan (7/22, 31.8%), followed by the United States (4/22, 18.2%) and Italy (3/22, 13.6%), with additional contributions from Scotland, Canada, Taiwan, France, Austria, and Germany (Table 2).

**Table 2:** Demographics of free functional muscle transfer after oncological resection (22 studies, 82 cases).

Characteristic	Findings
Publication years	1997–2025 (median ≈ 2016)
Cases per study	Median 1 (IQR 2.75); mean 2.83; max 17
Patient age	Median 55 (IQR 25); mean 50; min 9; max 82
Sex distribution	Male 46/82 (56.1%); Female 29/82 (35.4%); Not Reported 7/82 (8.54%)
Country of publication	Japan 7/22 (31.8%), USA 4/22 (18.2%), Italy 3/22 (13.6%), Scotland 2/22 (0.91%), Canada 2/22 (0.91%), Taiwan 1/22 (4.55%), France 1/22 (4.55%), Austria 1/22 (4.55%), Germany 1/22 (4.55%)

Abbreviations: IQR, Interquartile Range.

### Donor muscle and diagnosis categories

**Figure 2:**
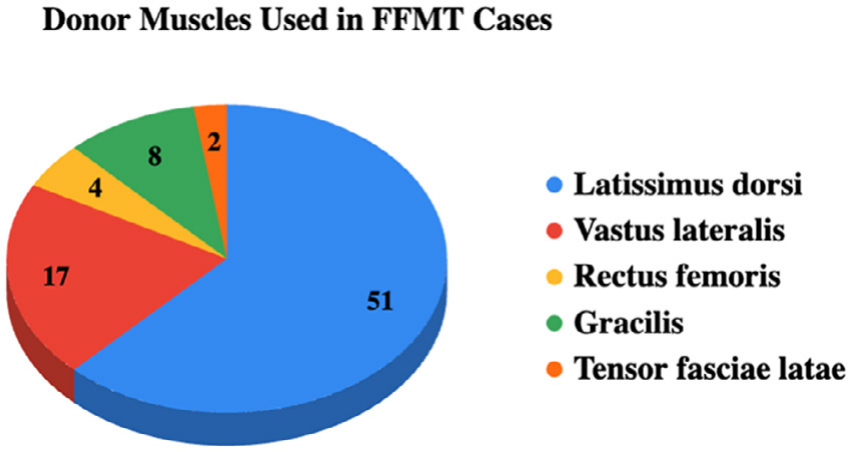
Distribution of donor muscles used in free functional muscle transfer cases following oncological resection.

The latissimus dorsi was the most frequently utilized donor muscle (51 cases; 62.2%), followed by vastus lateralis (17 cases; 20.7%). Less common donors included gracilis (9.8%), rectus femoris (4.9%), and tensor fasciae latae (2.4%) (Figure 2). Over time, vastus lateralis use increased, from none prior to 2014 to 14 cases (40%) in 2021–2025, while latissimus dorsi remained a dominant donor across all publication periods (Table 3).

**Table 3:** Donor muscle and era trends after oncological resection (82 cases).

Donor muscle used	≤2014	2015–2020	2021–2025
Latissimus dorsi	31	2	18
Gracilis	6	1	1
Tensor fasciae latae	2	0	0
Rectus femoris	2	0	2
Vastus lateralis	0	3	14
Total cases per era	41	6	35

**Figure 3:**
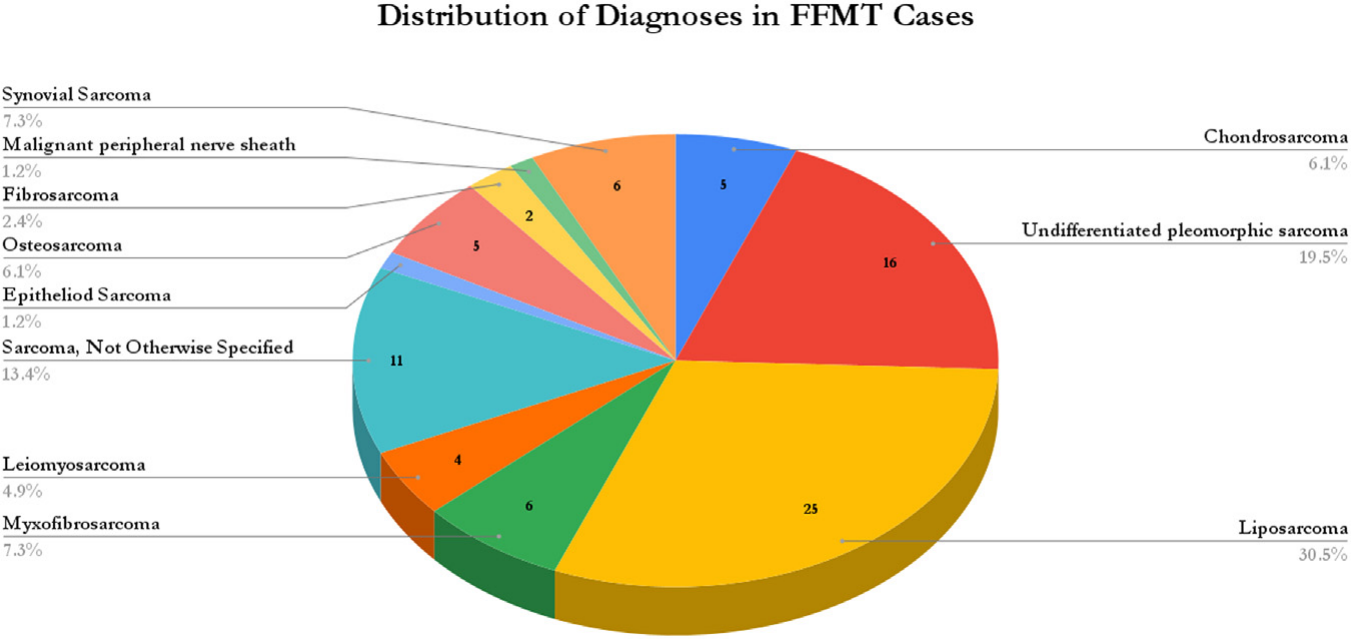
Distribution of diagnoses in free functional muscle transfer cases. Abbreviations: UPS, Undifferentiated Pleomorphic Sarcoma; Malignant PNST, Malignant Peripheral Nerve Sheath Tumor.

Across tumor histologies, liposarcoma was most common (30.5%), followed by undifferentiated pleomorphic sarcoma (19.5%) and unspecified sarcomas (13.4%). Other histologies included synovial sarcoma and myofibrosarcoma (each 7.3%), osteosarcoma and chondrosarcoma (each 6.1%), leiomyosarcoma (4.9%), fibrosarcoma (2.4%), and rare entities such as epithelioid sarcoma and malignant peripheral nerve sheath tumor (1.2% each) (Figure 3).

### Overall outcomes

#### Overall strength outcomes

Functional strength outcomes (MRC/MMT grades) demonstrated moderate-to-strong recovery overall, although reporting was incomplete (Table 4). Among all included cases, 43.9% (36/82; 95% CI 33.6–54.7%) achieved an average strength outcome in the >3 to ≤4 range, while 15.9% (13/82; 95% CI 9.5–25.3%) achieved >4 to ≤5 strength and an additional 15.9% (13/82; 95% CI 9.5–25.3%) fell within the >2 to ≤3 range. Strength outcomes were not consistently reported, with 14.63% (12/82; 95% CI 8.6–23.6%) of cases lacking extractable MRC/MMT.

**Table 4:** Average MRC/MMT grade range for all cases (*n* = 82).

Average MRC/MMT grade range	Proportion of cases	Rate (%)	95% CI
>4 and ≤5	13/82	15.90%	(9.5%, 25.3%)
>3 and ≤4	36/82	43.90%	(33.6%, 54.7%)
>2 and ≤3	13/82	15.90%	(9.5%, 25.3%)
≤2	8/82	9.76%	(5.0%, 18.0%)
Not reported	12/82	14.63%	(8.6%, 23.6%)

#### Strength outcomes by donor muscle

When stratified by donor muscle, average MRC/MMT grade was 3.55 (95% CI 3.31–3.79) for latissimus dorsi and 3.60 (95% CI 2.92–4.28) for gracilis, with reporting completeness of 90.2% and 62.5%, respectively. Vastus lateralis transfers demonstrated higher mean strength (4.21; 95% CI 3.61–4.82; reporting completeness 82.4%) compared with latissimus dorsi (3.55), with a statistically significant difference (*p* < 0.05). Rectus femoris transfers showed similar strength (4.17; 95% CI 2.28–6.06; reporting completeness 75%), while tensor fasciae latae transfers were rarely reported (*n* = 2) but demonstrated high average strength (4.5). However, several donor muscle subgroups were small (tensor fasciae latae *n* = 2, rectus femoris *n* = 4, gracilis *n* = 8), resulting in wide confidence intervals and unstable estimates. Accordingly, these comparisons should be interpreted as descriptive rather than evidence of functional superiority (Table 5).

**Table 5:** Average MRC/MMT grade stratified by donor muscle.

Donor muscle used	Total number of cases	Average MRC/MMT grade	95% CI	Range	Reporting completeness (Cases)
Latissimus dorsi	51	3.55	(3.31, 3.79)	2–5	46/51 (90.2%)
Gracilis	8	3.6	(2.92, 4.28)	3–4	5/8 (62.5%)
Tensor fasciae latae	2	4.5	(−1.85, 10.85)	4–5	2/2 (100%)
Rectus femoris	4	4.17	(2.28, 6.06)	3.5–5	3/4 (75%)
Vastus lateralis	17	4.21	(3.61, 4.82)	2–5	14/17 (82.4%)

Abbreviations: MRC, Medical Research Council; MMT, Manual Muscle Testing; CI, Confidence Interval.

#### Strength outcomes by reinnervation strategy

##### Neurorrhaphy

Of the 82 cases included, 80 (97.6%) utilized end-to-end neurorrhaphy, while only 2 cases (2.4%) from a single study (Frey et al.^21^) employed end-to-side neurorrhaphy, described as a novel technique. The average MRC/MMT grade for end-to-side neurorrhaphy was 3.25, with complete reporting (2/2; 100%). The average MRC/MMT for end-to-end neurorrhaphy was 3.76, with strength outcomes reported in 68 of 80 cases (85.0%) (Table 6). However, direct comparisons between the two neurorrhaphy strategies should not be drawn due to small sample size in the end-to-side group.

**Table 6:** Average MRC/MMT and complication rate stratified by neurorrhaphy technique.

Neurorrhaphy strategy used	Number of cases	Average MRC/MMT grade	95% confidence interval	Reporting completeness	Complication rate	95% confidence interval	Reporting completeness
End-to-end	80	3.76	(3.58, 3.94)	68/80 (85.0%)	16/58	(17.8%, 40.1%)	58/80 (72.5%)
End-to-side	2	3.25	(−3.10, 9.60)	2/2 (100%)	0/2	(0.0%, 65.8%)	2/2 (100%)

##### Donor nerve

Thoracodorsal nerve transfers were the most common (*n* = 49) and demonstrated an average MRC score of 3.53 (95% CI 3.29–3.77) with high reporting completeness (91.8%). Femoral nerve transfers (*n* = 22) showed higher average strength (4.22; 95% CI 3.86–4.58) but lower reporting completeness (68.2%). Obturator nerve transfers (*n* = 8) demonstrated moderate strength (3.60; 95% CI 2.92–4.28) with incomplete reporting (62.5%), while superior gluteal nerve transfers (*n* = 2) showed higher mean strength (4.50) with wide confidence intervals due to small sample size. One case with an unspecified donor nerve lacked reported strength outcomes. Functional outcomes by donor nerve are summarized in Table 7.

**Table 7:** Average MRC/MMT grade stratified by donor nerve used.

Donor nerve used	Number of cases	Average MRC/MMT grade	95% confidence interval	Reporting completeness
Femoral nerve	22	4.22	(3.86, 4.58)	15/22 (68.2%)
Thoracodorsal nerve	49	3.53	(3.29, 3.77)	45/49 (91.8%)
Obturator nerve	8	3.6	(2.92, 4.28)	5/8 (62.5%)
Superior gluteal nerve	2	4.5	(−1.85, 10.85)	2/2 (100.0%)
Not reported	1	NR	-	0/1 (0.0%)

Abbreviations: NR, Not Reported.

##### Recipient nerve

The femoral nerve was the most common recipient (*n* = 52), with an average MRC score of 3.81 (95% CI 3.59–4.03) and high reporting completeness (86.5%). Sciatic nerve transfers (*n* = 14) demonstrated similar outcomes (3.57; 95% CI 3.12–4.02) with complete strength reporting. Smaller subgroups—including radial, deep peroneal, axillary, and tibial nerves—demonstrated comparable strength (means ∼3.5–4.5) but with wider confidence intervals due to limited sample sizes. Strength outcomes were not reported for median and superior gluteal nerve cases. Functional outcomes stratified by recipient nerve are summarized in Table 8.

**Table 8:** Average MRC/MMT grade stratified by recipient nerve used.

Recipient nerve used	Number of cases	Average MRC/MMT grade	95% confidence interval	Reporting completeness
Axillary nerve	2	4.5	(3.23, 5.77)	2/2 (100%)
Deep peroneal nerve	3	3.5	(1.78, 5.22)	2/3 (66.67%)
Femoral nerve	52	3.81	(3.59, 4.03)	45/52 (86.54%)
Median nerve	1	NR	-	0/1 (0%)
Radial nerve	5	3.5	(2.52, 4.48)	4/5 (80.0%
Sciatic nerve	14	3.57	(3.12, 4.02)	14/14 (100%)
Superior gluteal nerve	1	NR	-	0/1 (0%)
Tibial nerve	2	3.75	(2.48, 5.02)	2/2 (100%)
Not reported	2	3	-	1/2 (50%)

##### Donor-recipient nerve pairings

**Figure 4:**
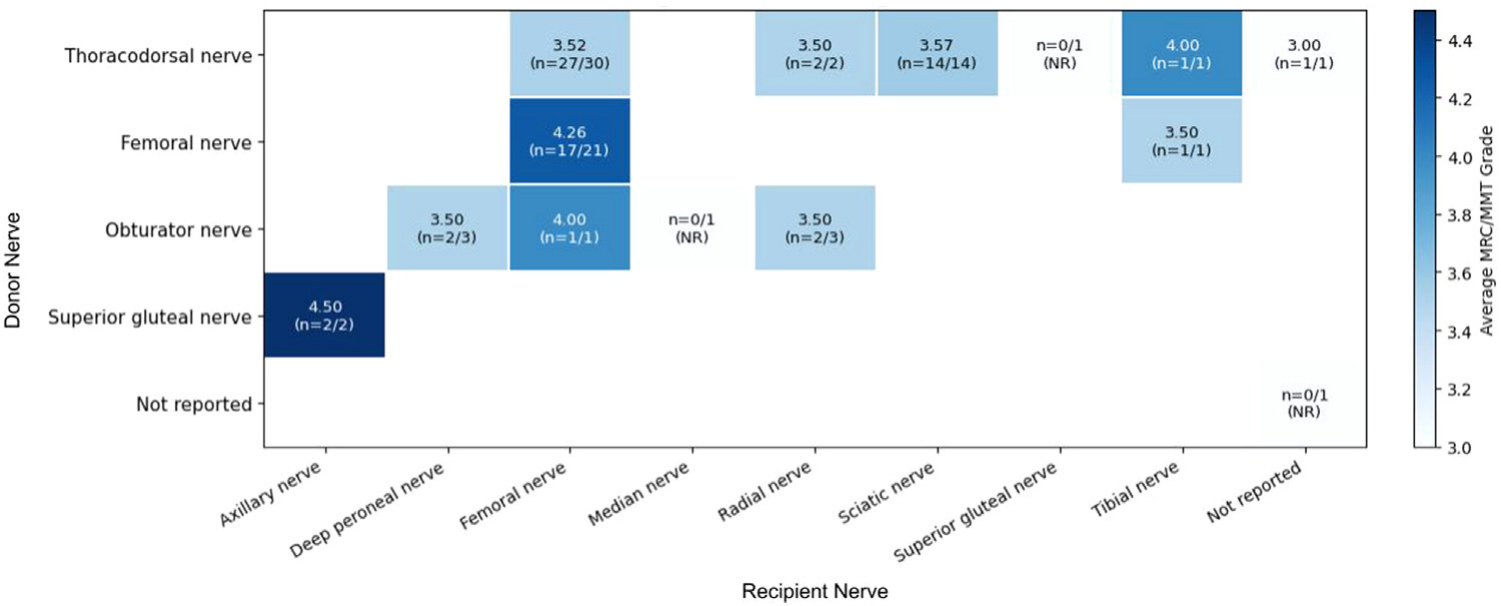
Donor-recipient motor nerve matrix with reporting outcomes and average MRC/MMT grade. Top number = Average MRC/MMT grade. n = number of donor-recipient nerve pairings reporting MRC/MMT grade/total number of specific donor-recipient nerve pairings in included articles. Abbreviations: NR, Not reported.

The most common pairing was thoracodorsal-to-femoral (*n* = 30), with an average MRC/MMT of 3.52 and high reporting completeness (90%). Thoracodorsal-to-sciatic transfers (*n* = 14) showed similar outcomes (3.57) with complete reporting, while other thoracodorsal pairings (thoracodorsal-to-radial and thoracodorsal-to-tibial) were less frequent and thus had wider confidence intervals. Among other combinations, femoral-to-femoral transfers (*n* = 21) demonstrated the highest average strength (4.26; reporting completeness 80.95%). Obturator nerve pairings showed variable outcomes across recipient nerves, while superior gluteal-to-axillary transfers (*n* = 2) demonstrated strong recovery (4.50). Overall, functional recovery was observed across a range of donor–recipient combinations, although many pairings were limited by small sample sizes or incomplete reporting (Figure 4).

#### Muscle survival

Among cases explicitly reporting flap survival, 77 of 77 flaps were successful, yielding a pooled survival rate of 100% (95% CI 95.3–100.0%). Reporting completeness for the survival endpoint was 93.9% at the case level (77/82) and 90.9% at the study level (20/22) (Table 9).

**Table 9:** Flap survival, complication rate, and reporting completeness.

Outcome	Numerator/Denominator	Rate (%)	95% CI
Flap survival	77/77	100%	(95.3%, 100.0%)
Any complication	16/60	26.67%	(17.1%, 39.0%)
Survival endpoint reporting completeness (cases)	77/82	93.90%	(86.5%, 97.4%)
Survival endpoint reporting completeness (studies)	20/22	90.90%	(72.2%, 97.5%)
Complication endpoint reporting completeness (cases)	60/82	73.20%	(62.8%, 81.6%)
Complication endpoint reporting completeness (studies)	19/22	86.36%	(66.7%, 95.3%)

Abbreviations: CI, Confidence Interval.

#### Overall complications

For complications, reporting completeness was 73.20% at the case level (60/82) and 86.36% at the study level (19/22). Of the cases explicitly reporting on postoperative complications, 16 of 60 cases experienced at least one adverse event, corresponding to a pooled complication rate of 26.67% (16/60; 95% CI 17.1–39.0%) (Table 9). To assess the impact of missing complication data, a best-case/worst-case sensitivity analysis was performed. Assuming no complications among the 22 cases without reported complication status yielded a minimum estimated complication rate of 19.5% (16/82), whereas assuming complications in all 22 unreported cases yielded a maximum estimated complication rate of 46.3% (38/82). Thus, the observed reported complication rate of 26.7% (16/60) should be interpreted within this broader plausible range.

#### Complications and prior therapy

Among cases with documented prior therapy (i.e., radiation therapy or chemotherapy), 12 of 37 cases (32.4%) experienced at least one postoperative complication (95% CI 19.6–48.5%), with complication status reported for 37 of 56 cases (66.1%) in this subgroup. In contrast, among cases without prior therapy, 3 of 15 cases (20.0%) experienced a complication (95% CI 7.0–45.2%), with complication reporting completeness of 83.3% (15/18). Cases in which therapy status was not reported had 0 complications among 7 cases (0.0%; 95% CI 0.0–35.4%), with complete complication reporting (7/7, 100%). Although the complication rate appeared higher among cases with prior radiation or chemotherapy compared with those without prior therapy (32.4% vs. 20.0%), this difference was not statistically significant (Fisher’s exact test, *p* ≈ 0.38). Table 10 summarizes complication rates stratified by the presence of prior radiation therapy or chemotherapy.

**Table 10:** Case-level complication rates stratified by presence of radiation therapy.

	Complication rate	95% confidence interval	Complication rate reporting completeness
Presence of prior radiation therapy or chemotherapy	12/37 (32.43%)	(19.6%, 48.5%)	37/56 (66.07%)
Absence of prior radiation therapy or chemotherapy	3/15 (20.0%)	(7.0%, 45.2%)	15/18 (83.33%)
Therapy status not reported	0/7 (0.0%)	(0.0%, 35.4%)	7/7 (100.0%)

#### Complications by donor muscle

Complication and recurrence rates varied by donor muscle. Among cases with complication reporting, latissimus dorsi transfers had complications in 8/35 cases (22.9%), *gracilis* in 1/8 (12.5%), and vastus lateralis in 4/12 (33.3%), the highest observed rate. No complications were reported for tensor fasciae latae (0/2) or rectus femoris (0/2) in available cases. Reporting completeness ranged from 50% for rectus femoris to 100% for gracilis and tensor fasciae latae, with intermediate completeness for latissimus dorsi (68.6%) and vastus lateralis (70.6%). Reported complications were primarily wound-related (infection, seroma, delayed healing, breakdown), with less frequent events including femur fracture, lymphedema, venous thrombosis, and skin necrosis (Table 11).

**Table 11:** Complications and tumor recurrence stratified by donor muscle.

Donor muscle used	Complications (*n*/*N*)	95% CI	Reporting completeness	Reported complications phenotypes	Tumor recurrence	Reporting completeness (Cases)
Latissimus dorsi	10/36 (27.78%)	(15.9%, 44.0%)	36/51 (70.1%)	Infection, femur fracture, delayed wound healing, seroma, lymphedema, venous thrombus	3/48 (6.25%)	48/51 (94.1%)
Gracilis	1/8 (12.5%)	(2.2%, 47.1%)	8/8 (100%)	Skin necrosis	0/7 (0%)	7/8 (87.5%)
Tensor fasciae latae	0/2 (0%)	(0.0%, 65.7%)	2/2 (100%)	None	0/2 (0%)	2/2 (100%)
Rectus femoris	0/2 (0%)	(0.0%, 65.7%)	2/4 (50%)	None	0/3 (0%)	3/4 (75%)
Vastus lateralis	5/12 (33.30%)	(3.61, 4.82)	12/17 (70.6%)	Infection, seroma, delayed wound healing, wound breakdown	1/13 (7.69%)	13/17 (76.5%)

Abbreviations: CI, Confidence Interval.

#### Complications by reinnervation strategy

##### Neurorrhaphy

End-to-end neurorrhaphy, the predominant technique in included studies, demonstrated a complication rate of 27.6% (16/58; 95% CI 17.8–40.1%), with reporting completeness of 58/80 (72.5%). In contrast, no complications were reported among the limited cases utilizing end-to-side neurorrhaphy (0/2; 95% CI 0.0–65.8%), with complete reporting (2/2; 100.0%). The wide confidence interval for the end-to-side group reflects the small sample size. Complication rates stratified by neurorrhaphy technique are presented in Table 6.

##### Donor and recipient nerve

Complication rates varied by both donor and recipient nerve, although interpretation is limited by incomplete reporting and small subgroup sizes. Among donor nerves, thoracodorsal transfers demonstrated the highest observed complication rate (76.5%, 26/34; 95% CI 60.8–87.1%) with limited reporting completeness (57.6%), while femoral nerve transfers showed lower rates (40.0%, 6/15; 95% CI 19.8–64.3%) and moderate completeness (68.2%). Obturator nerve transfers were associated with fewer complications (12.5%, 1/8; 95% CI 2.2–47.1%) with complete reporting, and superior gluteal nerve transfers reported no complications (0/2), though sample sizes were small; one case with an unspecified donor nerve reported a complication (1/1). Among recipient nerves, the most common femoral nerve demonstrated a complication rate of 30.8% (12/39; 95% CI 18.6–46.4%) with 75.0% reporting completeness, while deep peroneal and sciatic nerve transfers showed similar rates (∼33.3%) with wide confidence intervals and lower completeness for sciatic cases. No complications were reported for axillary, median, radial, superior gluteal, or tibial nerve transfers, although these findings are based on very small cohorts. Overall, complication rates varied across recipient nerve groups, with interpretation limited by incomplete reporting and small subgroup sizes (Table 12).

**Table 12:** Complication rates stratified by donor and recipient nerve used.

Donor nerve used	Complication rate	95% confidence interval	Reporting completeness
Femoral nerve	6/15 (40.0%)	(19.8%, 64.3%)	15/22 (68.2%)
Thoracodorsal nerve	26/34 (76.5%)	(60.8%, 87.1%)	34/59 (57.6%)
Obturator nerve	1/8 (12.5%)	(2.2%, 47.1%)	8/8 (100.0%)
Superior gluteal nerve	0/2 (0.0%)	(0.0%, 65.8%)	2/2 (100.0%)
Not reported	1/1 (100.0%)	(20.7%, 100.0%)	1/1 (100.0%)

Recipient nerve used	Complication rate	95% confidence interval	Reporting completeness
Axillary nerve	0/2 (0.0%)	(0.0%, 65.8%)	2/2 (100.0%)
Deep peroneal nerve	1/3 (33.3%)	(6.1%, 79.2%)	3/3 (100.0%)
Femoral nerve	12/39 (30.8%)	(18.6%, 46.4%)	39/52 (75.0%)
Median nerve	0/1 (0.0%)	(0.0%, 79.3%)	1/1 (100.0%)
Radial nerve	0/5 (0.0%)	(0.0%, 43.4%)	5/5 (100.0%)
Sciatic nerve	2/6 (33.3%)	(9.7%, 70.0%)	6/14 (42.9%)
Superior gluteal nerve	0/1 (0.0%)	(0.0%, 79.3%)	1/1 (100.0%)
Tibial nerve	0/2 (0.0%)	(0.0%, 65.8%)	2/2 (100.0%)
Not reported	1/1 (100%)	-	1/2 (50%)

#### Tumor recurrence

Tumor recurrence was uncommon across donor types, occurring in 3/48 latissimus dorsi cases (6.25%) and 1/13 vastus lateralis cases (7.69%), while no recurrences were reported in gracilis (0/7), tensor fasciae latae (0/2), or rectus femoris transfers (0/3) among cases with recurrence reporting. Recurrence reporting completeness was generally high, ranging from 75.0% (3/4) for rectus femoris to 100% for tensor fasciae latae, and exceeded 90% for latissimus dorsi (48/51, 94.1%) (Table 11).

## Discussion

This PRISMA-guided systematic review demonstrates that free functional muscle transfer (FFMT) can be a highly reliable reconstructive strategy for functional limb salvage after oncologic extremity resection, even across a heterogeneous and largely case-based evidence base. Across 22 studies (82 cases), no total flap losses were reported among cases with explicit survival reporting; however, this finding should be interpreted with caution. The included literature consisted primarily of case reports and small series, which are inherently susceptible to publication bias, and survival data were unavailable in 6.1% of cases, further limiting the certainty of this estimate. As such, the observed 100% survival rate likely reflects reported outcomes rather than a true population-level estimate of flap viability.

The literature suggests that some surgeons may be hesitant to perform limb reconstruction in patients who have received radiotherapy or chemotherapy due to the known deleterious effects of these therapies on surgical tissues.^1^ Both treatments have been associated with tissue necrosis, fibrosis, vascular injury, and impaired wound healing, all of which may increase postoperative complication risk.^1^^,^^30^^,^^31^ In our analysis, however, we found no statistically significant difference in complication rates between patients with and without prior therapy (Fisher’s exact test, *p* ≈ 0.38), although rates were numerically higher in treated patients (32.4% vs. 20.0%). Incomplete reporting limits the strength of this comparison, but these findings suggest that FFMT can achieve reliable reconstruction even in the setting of prior oncologic treatment.

The review further suggests that functional recovery was generally clinically meaningful, with most patients achieving antigravity-to-against-resistance strength: 43.9% achieved average strength >3 to ≤4, and 15.9% achieved >4 to ≤5, although incomplete reporting limits certainty. Our results are consistent with prior literature demonstrating low flap loss (0.8%) and moderate functional recovery (mean MRC ∼3.78).^2^ These findings support FFMT as a pragmatic salvage option capable of restoring purposeful motion in limbs that would otherwise be preserved only anatomically.

Donor muscle selection clustered around a limited number of workhorse options, reflecting a balance between excursion, force generation, and reliability.^3^^,^^4^ The predominance of the latissimus dorsi in our dataset mirrors prior reports, likely due to its consistent pedicle anatomy and ability to address both coverage and motor restoration in a single stage.^2^^,^^32^ Vastus lateralis appeared more prominently in recent series, suggesting evolving reconstructive strategies. Although some subgroups (i.e., vastus lateralis) demonstrated higher average strength, these differences should be interpreted cautiously, as donor selection is confounded by defect characteristics and small sample sizes limit meaningful comparisons. The relatively lower strength of the gracilis subgroup in our review aligns with prior literature and may support its use in forearm reconstruction, where force demands are lower^2^; however, additional data on less frequently used donor muscles are needed for definitive comparison.

From a reconstructive standpoint, end-to-end neurorrhaphy remained the dominant and most consistently reported technique with favorable outcomes, whereas evidence for end-to-side neurorrhaphy was extremely limited. The slightly lower mean MRC/MMT in the end-to-side cohort should therefore be interpreted cautiously given the small sample size and wide confidence interval, precluding meaningful comparison between techniques. Donor and recipient nerve selection appeared to influence functional recovery, although these relationships are likely confounded by reconstructive goals and anatomy rather than reflecting intrinsic superiority of specific nerves. Common recipient nerves such as the femoral and sciatic demonstrated consistent moderate-to-strong recovery, while less frequently reported nerves yielded more variable estimates due to small sample sizes. Similarly, functional recovery was observed across a range of donor–recipient pairings, with commonly used combinations such as thoracodorsal-to-femoral and thoracodorsal-to-sciatic demonstrating positive reproducible outcomes. Overall, these findings suggest that appropriate donor-recipient matching and surgical context are more important than any single optimal nerve or technique.

Complication rates were nontrivial, with approximately one-quarter of reported cases experiencing postoperative adverse events, and best-case/worst-case sensitivity analyses suggesting a potential range up to nearly half when accounting for missing data. When stratified by donor muscle type, complication rates ranged from 12.5% (gracilis) to 33.3% (vastus lateralis). Wound-related complications predominated, emphasizing that the principal threat to successful outcomes is often not flap perfusion alone but the broader physiologic and wound-healing context of oncologic reconstruction.^33^ Less common but consequential events such as venous thrombosis and femur fracture were also reported in latissimus cohorts. While end-to-end neurorrhaphy appeared to have an acceptable but nontrivial complication rate, conclusions regarding end-to-side neurorrhaphy are limited by the extremely small sample size Likewise, donor and recipient nerve choices did not demonstrate a clear pattern of complication rates and likely reflect variation in case complexity, defect characteristics, or selection bias rather than true differences in risk.

Although oncologic outcomes were inconsistently reported, recurrence appears to be driven primarily by tumor biology, margin status, and adjuvant therapy rather than reconstructive technique.^3^^,^^34^ Still, reconstructive strategy intersects with oncologic care through wound healing and the ability to tolerate timely radiotherapy or chemotherapy. Free functional muscle transfers may also facilitate oncologic management by allowing wider resections to achieve negative margins while still preserving the potential for meaningful functional restoration.^3^ Therefore, modern sarcoma care is increasingly framed as a multidisciplinary effort to optimize both oncologic control and function, with expanding reconstructive options for both coverage and functional restoration.^35^

## Limitations and future research

The evidence base for oncologic FFMT is limited by small, heterogeneous case reports and retrospective series, with susceptibility to publication bias and selective reporting. While survival reporting was relatively complete, complication and functional outcomes were less consistently reported and variably defined. Functional measures were not standardized (e.g., MRC/MMT vs. DASH or MSTS), limiting comparability. Additionally, small donor muscle subgroups produced wide confidence intervals and unstable estimates, and donor comparisons are confounded by indication and anatomy, as flap selection is individualized. Accordingly, pooled estimates should be interpreted as descriptive rather than definitive benchmarks.

Within these limitations, FFMT appears to provide reliable flap viability and often meaningful strength recovery when major motor units are resected. Early multidisciplinary planning is critical, particularly for dead-space management, recipient selection, and rehabilitation integration. Future studies should prioritize standardized outcome definitions, consistent strength reporting, and complete functional data, with larger prospective or registry-based studies needed to enable more definitive comparisons.

## Conclusion

Free functional muscle transfer represents a critical advancement in modern oncologic limb-salvage surgery, extending reconstruction beyond soft-tissue coverage to restoration of active motor function after resection of major musculotendinous units. Across the available literature, FFMT demonstrates high flap reliability and frequently meaningful strength recovery, supporting its role as both a coverage and reanimation strategy when negative-margin resection would otherwise result in severe disability. Although the current evidence base is limited by small, heterogeneous series and variable reporting, the cumulative data suggest that—with careful patient selection, multidisciplinary planning, and structured rehabilitation—FFMT can preserve not only limb viability but also purposeful function. Continued standardization of outcomes and prospective data collection will be essential to refine technique selection and strengthen the evidence supporting this complex but impactful reconstructive approach.

## Funding

This research received no external funding.

## Declaration of competing interest

The authors declare no conflicts of interest.
